# Microbiome and Resistome Studies of the Lithuanian Baltic Sea Coast and the Curonian Lagoon Waters and Sediments

**DOI:** 10.3390/antibiotics13111013

**Published:** 2024-10-28

**Authors:** Greta Gyraitė, Marija Kataržytė, Rafael Picazo Espinosa, Greta Kalvaitienė, Eglė Lastauskienė

**Affiliations:** 1 Bioscience Institute, Life Science Center, Vilnius University, 10257 Vilnius, Lithuania; egle.lastauskiene@gf.vu.lt; 2 Marine Research Institute, Klaipeda University, 92295 Klaipėda, Lithuania; marija.katarzyte@ku.lt (M.K.); rafael.picazo.espinosa@ku.lt (R.P.E.); greta.kalvaitiene@ku.lt (G.K.)

**Keywords:** microbial community, antibiotic resistance genes, bathing waters

## Abstract

Background: the widespread use of antibiotics in human and veterinary medicine has contributed to the global challenge of antimicrobial resistance, posing significant environmental and public health risks. Objectives: this study aimed to examine the microbiome and resistome dynamics across a salinity gradient, analyzing water and sediment samples from the Baltic Sea coast and the Curonian Lagoon between 2017 and 2023. Methods: the composition of the water and sediment bacterial community was determined by Full-Length Amplicon Metagenomics Sequencing, while ARG detection and quantification were performed using the SmartChipTM Real-Time PCR system. Results: the observed differences in bacterial community composition between the Baltic Sea coast and the Curonian Lagoon were driven by variations in salinity and chlorophyll a (chl a) concentration. The genera associated with infectious potential were observed in higher abundances in sediment than in water samples. Over 300 genes encoding antibiotic resistance (ARGs), such as aminoglycosides, beta-lactams, and multidrug resistance genes, were identified. Of particular interest were those ARGs that have previously been detected in pathogens and those currently classified as a potential future threat. Furthermore, our findings reveal a higher abundance and a distinct profile of ARGs in sediment samples from the lagoon compared to water. Conclusions: these results suggest that transitional waters such as lagoons may serve as reservoirs for ARGs, and might be influenced by anthropogenic pressures and natural processes such as salinity fluctuation and nutrient cycling.

## 1. Introduction

The discovery of antimicrobial substances is considered one of the most important scientific achievements of the 20th century, revolutionizing human, veterinary medicine, and agriculture [1]. However, antibiotics have recently been identified as a new class of environmental pollutants due to their overuse and the bioaccumulation of their remaining substances, so-called antibiotic residues [2,3]. Antibiotic residues and their byproducts enter the environment with domestic, hospital, and industrial wastewater, such as pharmaceutical, aquaculture, livestock, and agricultural runoff [4]. According to Suzdalev [5] in 2017, four Lithuanian coastal municipalities released about 20 kg of antibiotics such as azithromycin, ciprofloxacin, clarithromycin, erythromycin, and sulfamethoxazole per year after wastewater treatment. The last three antibiotics were found in surface water samples from all investigated sampling sites of the Curonian Lagoon, the channel of Klaipeda Port, and the Tenžė river [5]. The release of antibiotics into the environment, due to their biological activity and persistence, may represent a new and significant change in environmental quality and raises concerns about the potential ecotoxicological effects on aquatic life, as well as the development of antimicrobial resistance in wildlife [6].

Even low levels of antibiotic residues in natural aquatic ecosystems can adversely affect the antimicrobial resistance (further—AMR) of many bacterial strains. Some bacteria may become superbugs, resistant to multiple antibiotics, or emerge as new pathogens by acquiring antibiotic resistance genes (ARGs) from “a collection of all the ARGs and their precursors in pathogenic and nonpathogenic bacteria” [7], so-called environmental resistome and pose an increasing threat to public health [8]. The European Centre for Disease Prevention and Control has indicated that in the EU/EEA, the annual number of deaths attributable to infections of antibiotic-resistant bacteria ranged from 30,730 in 2016 to 38,710 [9]. Gene sharing through the horizontal gene transfer via mobile genetic elements (MGEs), such as transposons, plasmids, and integrons, contributes significantly to the global spread of ARGs. It is, therefore, crucial to improve the global surveillance of evolutionary changes in the resistome and implement mitigation strategies for ARG spread [10,11].

The main reservoirs of ARGs are wastewater treatment plants, soil, air, and effluent-receiving waters [12], such as groundwater, surface water, or even drinking water. Oceans and seas serve as global reservoirs of antibiotic-resistant bacteria and ARGs [13]. In the Baltic Sea region, four different tetracycline resistance genes tetA, tetC, tetH, and tetM [14] and the resistome [15,16,17] have been identified but only in farmed fish feces and sediments from aquaculture farms and their surroundings. As a result, the impact of aquaculture as a potential source of antibiotic pollution has been discovered. However, these studies do not reveal the current ARG profiles of coastal waters and sediments of the Baltic Sea. In addition, coastal lagoons are known to accumulate antibiotic residues due to their long retention time [18] and may act as a reservoir for ARGs and MGEs [19,20]. Unfortunately, there is no knowledge about the antimicrobial profile of the microbiota of Baltic Sea estuaries or lagoons such as the Curonian lagoon, Europe’s largest eutrophic coastal lagoon. It receives about 90% of its water and nutrients, and thus pollutants, from the discharges of the Nemunas River catchment area [21]. Furthermore, it is essential to study the microbiome and resistome concerning environmental conditions such as temperature and eutrophication-related traits (e.g., chla), which may shape the bacterial community by providing needed nutrients [22] or salinity, which may define the fate of ARGs by reducing their abundance [23].

Thus, we aimed to assess the dynamics of water and sediment microbial communities and their correlation to key environmental drivers along the salinity gradient on the Baltic Sea coast and the Curonian Lagoon. This study also provide the first quantitative screening of the resistome profiles, including microbial contaminants, as well as genetic elements such as ARGs, MGEs, and other genes encoding resistance to various heavy metals and disinfectants.

## 2. Results

### 2.1. Bacterial Richness and General Community Composition on Phylum and Class Level

The Chao1 and Shannon indexes were used to analyze the microbial community diversity in the sample. The Chao1 and Shannon indexes of the water samples exhibited considerable variation, ranging from 251 and 6.88 in Kintai (2018) to 385 and 7.50 in Šventoji (2021). In contrast, the sediment samples demonstrated a wider range, with values spanning from 230 and 7.20, respectively, in Kintai (2023) to 451 and 8.48 in Melnragė (2023). The Chao1 and Shannon indexes can be found in Supplementary Material Table S1.

**Figure 1:**
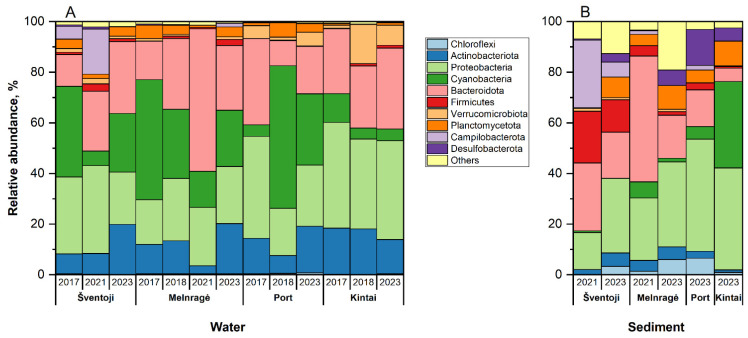
The relative abundances of the ten most prevalent bacterial phyla in the water (**A**) and sediment samples (**B**) were analyzed, with a minimum relative abundance of 1% in at least one sample. The remaining phyla, in addition to the ten most abundant phyla, are collectively represented by a class named “Others”.

The analysis of the water and sediment samples revealed that the ten phyla with the highest relative abundance of identified microorganisms were *Actinobacteriota*, *Proteobacteria*, *Cyanobacteria*, *Bacteroidota*, *Firmicutes*, *Verrumicrobiota*, *Planctomycetota*, *Campilobacterota*, *Chloroflexi*, and *Desulfobacterota* (Figure 1).

The most prevalent bacterial phyla in all water samples were *Actinobacteriota*, *Proteobacteria*, and *Cyanobacteria*. The cumulative relative abundance of these phyla varied considerably, from 40.81% in Melnragė (2021) to 82.57% in the Port area in 2018. The *Actinobacteriota* phylum exhibited a lower abundance in sediment samples than in water samples. Conversely, the relative abundance of *Proteobacteria* was approximately equivalent in water and sediment samples. The highest relative abundance of *Cyanobacteria* was observed in the Port area in 2018, while the lowest was observed in the Port area in 2023. In sediment samples, the relative abundance of *Cyanobacteria* exhibited variation, ranging from 0% in Šventoji (2023) to 34.14% in Kintai (2023). The relative abundance of *Bacteroidota* phylum bacteria in water samples varied, from 9.84% in the Port area in 2018 to 56.28% in Melnragė in 2021. In sediment samples, the range was even more pronounced, spanning from 5.28% in Kintai (2023) to 49.75% in Melnragė in 2021.

The *Firmicutes* were less frequently detected, with a relative abundance of less than 5% in the water samples. In contrast, in the sediment samples, particularly at the Šventoji site in 2021, the relative abundance reached 20.47%. A significant difference in the relative abundance of *Campilobacterota* was observed among the study sites (*p* < 0.05). It was most prevalent in both water (in 2017 and 2021) and sediment samples (in 2021 and 2023) at the Šventoji site. The *Verrucomicrobiota* phylum bacteria were present in higher abundance in the water samples from the Kintai site, with a relative abundance of 7.98% in 2023 and 15.45% in 2018. Conversely, they were less frequently detected in the remaining water and sediment samples, with a relative abundance of less than 5%. *Desulfobacterota* was absent from the water samples taken at the Kintai site. However, the most abundant phylum in the sediment samples from Kintai was *Desulfobacterota*, with a relative abundance of 5.26%, while in the Port area, it reached 14.19% in 2023. The relative abundance of the *Chloroflexi* phylum was higher in sediment samples (ranging from 1.2% to 5.6%) compared to water samples (with a minimum of less than 1%).

**Figure 2:**
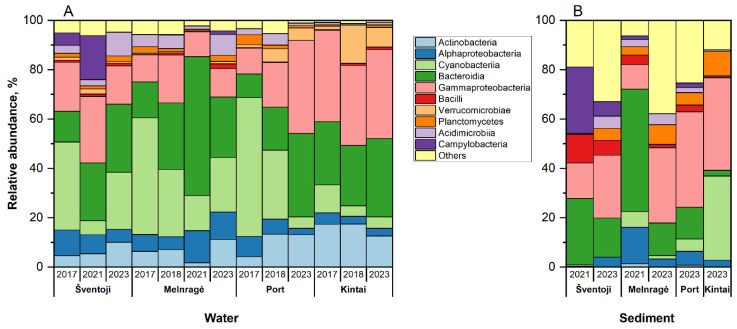
The relative abundances of the ten most prevalent bacterial classes in water (panel **A**) and sediment samples (panel **B**), with a minimum relative abundance of 1% in at least one sample. In addition to the ten most abundant, the remaining classes are collectively represented by a class named “Others”.

Twenty-four classes belonging to 14 phyla were identified in abundances of ≥1% in at least one sample. The relative abundance of the classes revealed that ten most abundant microorganism phyla in the samples were *Actinobacteria* (0–17.39%), *Alphaproteobacteria* (0.27–14.81%), *Cyanobacteria* (0.03–56.37%), *Bacteroidia* (2.29–56%), *Gammaproteobacteria* (9.92–38.68%), *Bacilli* (0–11.61%), *Verrucomicrobiae* (0–15.45%), *Planctomycetes* (0.04–9.76%), *Acidimicrobiia* (0.07–9.49%), and *Campylobacteria* (0–26.8%). The remaining classes were as follows: *Gammaproteobacteria* (9.92–38.68%), *Bacilli* (0–11.61%), *Verrucomicrobiae* (0–15.45%), *Planctomycetes* (0.04–9.76%), *Acidimicrobiia* (0.07–9.49%), *Campylobacteria* (0–26.8%), and others (0.79–37.89%) (Figure 2).

The most prevalent bacterial classes in all water samples were *Cyanobacteria*, *Bacteroidia*, and *Gammaproteobacteria*. The cumulative relative abundance of these classes ranged from 56.02% in Šventoji (2021) to 80.62% in Melnragė in 2018. The relative abundance of *Cyanobacteria* exhibited variation, with values ranging from 4.2% in Kintai (2018) to 56.36% in the Port area in 2017. In sediment samples, the relative abundance of *Cyanobacteria* ranged from 0.03% in Šventoji in 2023 to 34.13% in Kintai (2023).

The relative abundance of *Bacteroidia* was twofold higher in 2021 compared to 2023 in both water (from 56.28 to 24.55%) and sediment (49.75 to 13.23%) samples at the Melnragė site. The relative abundance of *Gammaproteobacteria* ranged from 10.12 to 37.68% in water samples and from 9.9 ± to 38.69% in sediment samples. Notably, the highest relative abundance in water samples was observed in the Port area in 2023.

The mean relative abundance of *Verrucomicrobiae* and *Acidimicrobiia* in water samples was comparable, with values of 3.23 ± 4.38% and 3.55 ± 2.92%, respectively. In contrast, the relative abundance of these organisms in sediment samples was approximately twofold lower, ranging from 0.15 ± 0.17% to 2.5 ± 1.95%, respectively. The relative abundance of *Plancomycetes* varied from 0.09 to 4.14% in water samples and from 0.04 to 9.76% in sediment samples. *Campylobacter’s* mean relative abundance was higher in sediment samples (6.00 ± 10.40%) than in water samples (1.93 ± 4.97%).

The mean relative abundance of *Actinobacteria* in water samples was 9.5 ± 5.08%, while in sediment samples, it was only 0.34 ± 0.54%. The highest relative abundance of *Actinobacteria* was observed in a water sample from Kintai in 2018. The mean relative abundance of *Alphaproteobacteria* in water and sediment samples exhibited a notable similarity, with values of 6.72 ± 3.26% and 5.09 ± 5.06%, respectively. In Šventoji (2017), the maximum relative abundance of *Bacilli* was observed (11.60%), with an average of 4.33 ± 4.06%. In contrast, the average relative abundance of *Bacilli* in water samples was only 0.66 ± 0.53%. A distinctive observation in the sediment samples from Šventoji in 2021 was the presence of genera typically associated with infection potential, including *Acinetobacter* and *Clostridium*. In contrast, the 2023 samples exhibited a prevalence of *Streptococcus*, *Helicobacter*, *Enterococcus*, and *Bacteroides*.

A statistically significant (*p* > 0.05) difference in the relative abundance of bacterial classes was observed between different years for *Cyanobacteria*, *Bacteroidia*, *Verrucomicrobiae*, *Acidimicrobiae*, and *Campylobacteria*. In contrast, the relative abundance of *Gammaproteobacteria*, *Planctomycetes*, and *Campylobacteria* differed significantly between sites. A PCoA analysis of the UniFrac weighted distances shows that the samples are segregated by sample type, with sediment samples distinct from water samples. Furthermore, the water samples from the Kintai sampling site are located away from the coastal sampling sites. Two water samples from 2021 occupy an intermediate position between water and sediment samples, possibly identifying a mixed community (Supplementary Figure S1).

### 2.2. Correlation Between the Ten Most Abundant Classes of Bacteria and Environmental Parameters

**Figure 3:**
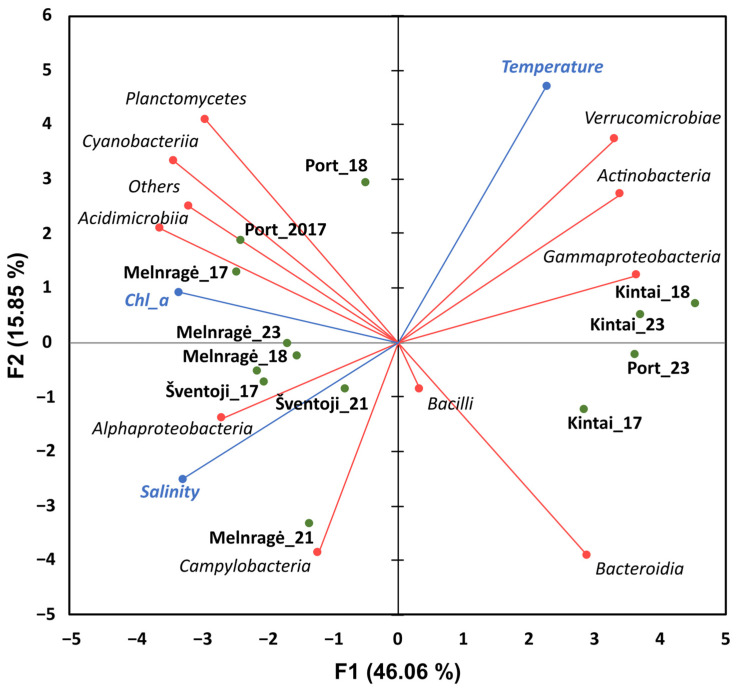
Principal component analysis (PCA) representing the correlation between the ten most abundant bacterial classes (red lines) in water and the physical (temperature and salinity) and biological (chl a) water parameters (blue lines) at all study sites, namely, Šventoji, Melnragė, the Port area, and Kintai, throughout the summer seasons of 2017, 2018, 2021, and 2023 (green dots).

Water temperature, salinity, and chl a concentration were recorded at the sampling sites in 2017, 2018, 2021, and 2023. The mean water temperature during the sampling was similar for all sampling sites, ranging from 17.85 ± 2.87 °C in Šventoji to 19.85 ± 2.56 °C in the Port area. Descriptive statistics of each parameter per sampling site are shown in Supplementary Table S3. Still, it differed significantly among years (*p* < 0.05). Conversely, salinity differed significantly between sampling sites (*p* < 0.05), while no differences were observed between years. PCA analysis applied to our dataset (10 most abundant bacterial classes, temperature, salinity, and chl a) showed that 61.91% of the total variance could be explained by components F1 = 46.06% and F2 = 15.85% (Figure 3).

Spearman correlation analysis showed a significant negative correlation between *Actinobacteria* (rS = −0.71, *p* < 0.05), *Gammaproteobacteria* (rS = −0.68, *p* < 0.05), and *Verrucomicrobiae* (rS = 0.71 *p* < 0.05) with salinity, whereas *Acidimicrobiae* (rS = 0.56, *p* < 0.05) was significantly positively correlated with salinity. *Verrucomicrobiae* was the only bacterial class with a significant positive correlation with temperature (rS = 0.74, *p* < 0.05). The relative abundance of *Actinobacteria* (rS = −0.59, *p* < 0.05) and *Gammaproteobacteria* (rS = 0.56, *p* < 0.05) correlated negatively with chl a, while *Cyanobacteria* (rS = 0.59, *p* < 0.05) and *Acidimicrobiia* (rS = 0.61, *p* < 0.05) correlated positively.

### 2.3. Profiles of Environmental Antibiotic Resistomes in Water and Sediment Samples of the Curonian Lagoon and Lithuanian Baltic Sea Coast

#### 2.3.1. Extended Profiles of Environmental ARGs, MGEs, MDRs, and Others

**Figure 4:**
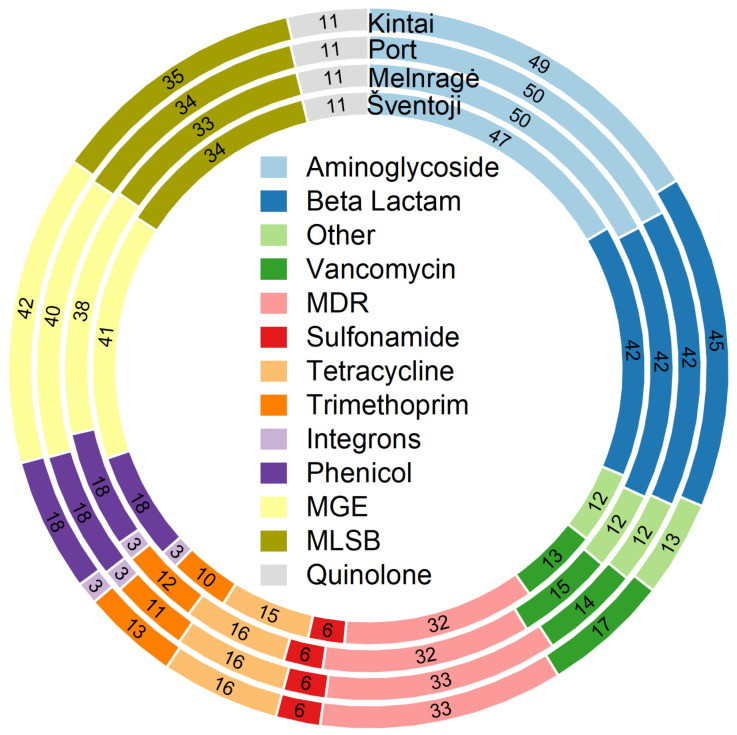
The diversity of antibiotic resistance genes detected in water and sediment pooled DNA samples from the Curonian Lagoon and the Baltic Sea in 2017, 2018, 2021, and 2023 at the Šventoji, Melnragė, Port, and Kintai sites.

The initial testing of four pooled DNA samples with a 384 ARG primer set yielded the following results: 284 genes coding for antibiotic resistance in Šventoji, 288 in Melnrage, 290 in the Port area, and 301 in Kintai (Figure 4).

Fifty percent of all identified resistance genes were found to belong to MDR and three distinct antibiotic groups: aminoglycosides, beta-lactams, and MLSB. The ARGs encoding resistance to aminoglycosides (n = 60) varied from 47 (78.33%) in Šventoji to 50 (83.3%) both in Melnragė and Port area. A total of 42 (77.78%) beta-lactam resistance genes were identified in the Šventoji, Melnragė, and Port samples, with a slightly higher number of genes detected in the Kintai sample (45, 83.33%). The MLSB-encoding genes ranged from 33 (70.21%) in Melnragė to 35 (74.47%) in Kintai. The number of MDR genes varied from 32 (82.05%) in Šventoji and Melnragė to 33 (84.62%) in Port and Kintai.

The percentage of detected vancomycin, tetracycline, trimethoprim, phenicol, sulfonamide, quinolone, and other ARGs varied from 29.93% in Šventoji to 31.24% in Melnragė. Three integron genes were observed across all sampling sites, while the number of MGE genes ranged from 38 (79.19%) in Melnragė to 42 (87.50%) in Kintai. More details about the detected genes in different sampling sites can be found in Supplementary Table S2.

**Figure 5:**
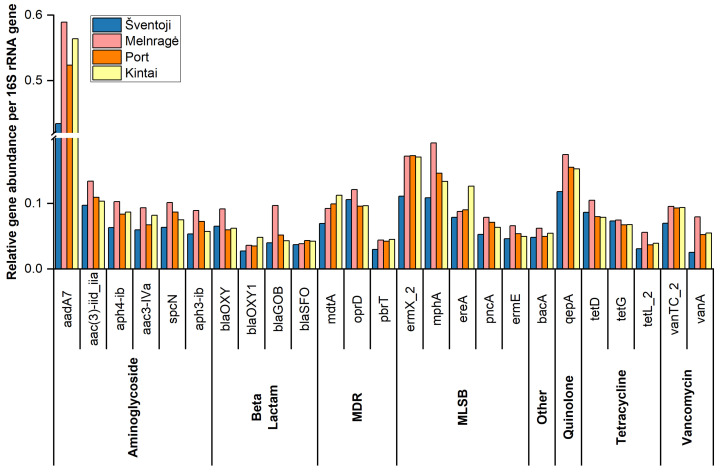
The relative abundance of the 25 most abundant antibiotic resistance genes in pooled samples of water and sediment from the Curonian Lagoon and the Baltic Sea, collected in 2017, 2018, 2021, and 2023 at the Šventoji, Melnragė, Port, and Kintai sites.

Of the 25 most prevalent ARGs identified in the pooled Šventoji, Melnragė, Port area, and Kintai sites, six encoded resistance to aminoglycosides (*aadA7*, *aac(3)-iid_iia*, *aph4-ib*, *aac3-Iva*, *spcN*, and *aph3-ib*), five to MLSB (*ermX_2*, *mphA*, *ereA*, *pncA*, and *ermE*), beta-lactams (*blaOXY*, *blaOXY1*, *blaGOB*, and *blaSFO*), multidrug resistance (*mdtA*, *oprD*, and *pbrT*), tetracyclines (*tetD*, *tetG*, and *tetL_2*), vancomycin (*vanTC_2* and *vanA*), quinolones (*qepA*), and other antibiotics (*bacA*). The number of genes encoding resistance to each antibiotic is shown in Figure 5.

The distribution of relative gene abundance per 16S rRNA gene was approximately equal across the study sites. The highest relative gene abundance per 16S rRNA gene across all study sites was observed for the aminoglycoside *aadA7* gene. Genes not included in the 25 most abundant ARG list but of high importance, such as the *mcr1* gene encoding resistance to colistin, the S1 and S3 integron class genes, and transposons of mobile genetic elements, were identified in all study sites (Supplementary Table S2).

#### 2.3.2. Detection of Veterinary and Clinical Importance ARGs and MDRs

**Figure 6:**
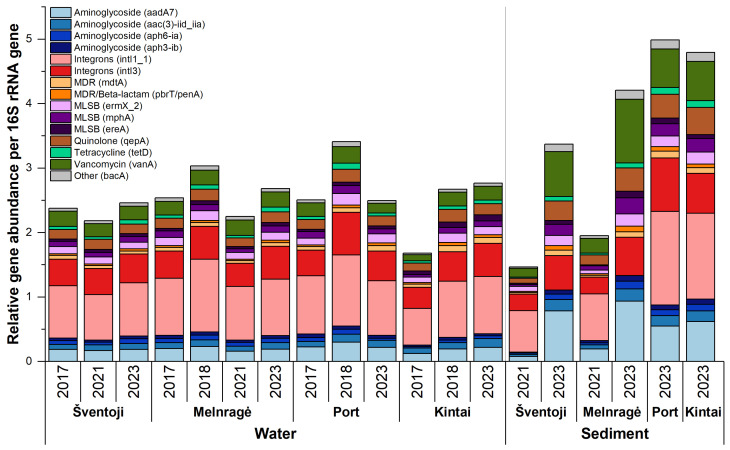
The cumulative relative abundance of the 15 most abundant antibiotic resistance genes in water and sediment samples from the Curonian Lagoon and the Baltic Sea, collected in 2017, 2018, 2021, and 2023 at the sites of Šventoji, Melnragė, Port, and Kintai.

In total, 80 ARGs were selected for comprehensive examination across multiple study sites and periods. A minimum of 60 ARGs (75.94%) were detected in the sediment sample from the Melnragė site in 2023, while a maximum of 77 ARGs (97.47%) was observed in the sediment samples from the Port area in the same year. The average detection rate in water samples was 75 ARGs out of the 80 tested. The 15 most abundant ARGs were identified in relative abundance in water and sediment samples from the Curonian Lagoon and the Baltic Sea (Figure 6).

The most prevalent of these were the four aminoglycoside group genes (*aadA7*, *aac(3)-iid_iia*, *aph6-ia*, and *aph3-ib*), with the *aadA7* gene being the most abundant. Its average proportion to the 16S rRNA gene was 0.20 ± 0.04 gene copies in water samples and 0.52 ± 0.33 gene copies in sediment samples. There was a significant difference in the relative abundance of the *aac(3)-iid_iia*, *aph6-ia*, and *aph3-ib* aminoglycosides between the sediment and water samples (*p* < 0.05, n = 12).

The 15 most abundant ARGs consisted of three macrolides, lincosamides, and streptogramins group genes (*ermX_2*, *ereA*, and *mphA*), of which the most abundant was the *ermX_2* gene, with an average of 0.12 ± 0.02 gene copies in proportion to the 16S rRNA gene in water and 0.13 ± 0.5 gene copies in sediment samples. A significant difference was observed in the relative abundance of the genes *ermX_2* and *mphA* among the sediment and water samples (*p* < 0.05, n = 12).

The genes *qepA*, *tetD*, *vanA*, and *bacA*, which are part of the quinolone, tetracycline, vancomycin, and other antibiotics group, respectively, were also identified. The proportion of the quinolone gene *qepA* in relation to the 16S rRNA gene in water samples ranged from 0.12 to 0.20 gene copies, while in sediment samples, it varied from 0.07 to 0.41 gene copies. The highest observed value was in sediment samples from Kintai in 2023. The relative abundance of the tetracycline gene *tetD* exhibited a range of 0.03 to 0.09 in water samples and 0.01 to 0.10 gene copies in sediment samples, with the latter measured in proportion to the 16S rRNA gene. The mean relative abundance of the vancomycin gene *vanA* in water samples was 0.20 ± 0.04 gene copies in proportion to the 16S rRNA gene, while in sediment samples, it was 0.54 ± 0.31. The highest concentration of the *vanA* gene, at 0.99 gene copies, was observed in sediment samples from the Melnragė site in 2023.

Of the 15 most prevalent resistance genes, three MDR genes (*mdtA*, *pbrT*, and *penA*) were identified. The mean relative abundance of *mdtA* was comparable between water (0.06 ± 0.01 gene copies in proportion to the 16S rRNA gene) and sediment samples (0.06 ± 0.03). The *pbrT* gene, which encodes a lead-importing protein, was identified among the 15 most abundant genes in water samples from all study sites but not in sediment samples. Conversely, the *penA* gene was identified in greater abundance in sediment samples than in water samples.

Genes that are considered to be related to anthropogenic pollution (e.g., *intI*, *sul1*, and *blaOXA*), as well as genes of clinical and veterinary relevance (beta-lactams, quinolones, and vancomycin), were identified in all of the studied sites. However, they did not fall into the 15 most abundant ARGs list. Moreover, a number of the identified ARGs exhibited a unique appearance. Trimethoprim (*dfrA1_1*), phenicol (*catB9*), beta-lactam (*blaTEM*), *nimE* associated with other antibiotics, quinolone (*qnrS_1*), and MLSB (*vatE_2*) were observed only in sediment samples collected in the Port area and Kintai in 2023. Two aminoglycoside ARGs were particularly notable in the water samples: *aph(3*″)-*ia* was exclusively present in coastal sampling sites Šventoji and Melnragė and was detected once in the Port area. However, it was not observed in the Curonian Lagoon sampling sites. Conversely, *aph3-viia* was exclusively observed in the Curonian Lagoon sampling sites and was not detected in the coastal sampling sites.

## 3. Discussion

The use of antibiotics to treat bacterial infections in humans or animals can result in adverse effects and contribute to the emergence of antibiotic-resistant bacteria [12]. The term “antibiotic resistance” refers to the ability of pathogens to evade the effects of antibiotics that are designed to kill them [24]. It is essential to investigate antimicrobial resistance profiles in aquatic environments to gain insight into the dissemination of antibiotic-resistant pathogens. By analyzing the distribution and concentration of antimicrobial-resistant genes, it is possible to identify contamination from human or animal waste, agricultural runoff, or industrial discharges [13].

Our study investigates the dynamics of microbial communities under the salinity gradient, temperature, and anthropogenic pressure and the distribution of over 300 genes encoding resistance to various antibiotics in sediment and water samples from four sites along the Baltic Sea coast of Lithuania and the Curonian Lagoon over six years.

### 3.1. Dominance and Variation of Bacterial Phyla and Classes

The variations in bacterial community composition between the Baltic Sea coast and the Curonian Lagoon sampling sites were found to be associated with salinity and chl a concentration. The abundance of *Acidimicrobiae* and *Alphaproteobacteria* was higher in brackish water sampling sites, such as Šventoji and Melnragė. Additionally, *Acidimicrobiia* were found in higher abundances in Melnragė and Port area sampling sites with higher salinity and chl a concentration, which is likely due to the transport of the water from the Curonian Lagoon [25]. *Actinobacteria*, *Gammaproteobacteria*, and *Verrucomicrobiae* negatively correlated with salinity, with higher abundances observed in the Kintai site and port area.

The dominance of the phyla *Actinobacteriota* (most abundant classes of *Actinobacteria* and *Acidimicrobiia*), *Proteobacteria* (classes *Alphaproteobacteria* and *Gammaproteobacteria*), and *Cyanobacteria* (class *Cyanobacteria*) in water samples underlines their crucial role in aquatic ecosystems, especially in nutrient cycling and primary production. *Cyanobacteria* compete with *Actinobacteriota* and *Proteobacteria* for nutrients, leading to microbial dysbiosis. *Actinobacteriota*, as well as *Proteobacteria*, with many well-known genera such as *Acinetobacter*, *Enterococcus*, *Escherichia*, and *Pseudomonas*, are known as ARG-carrying bacteria [26], possessing mobile genetic elements such as transposons and integrons; therefore, inhibiting their growth by cyanobacterial blooms is expected to reduce ARG density in freshwater ecosystems [27].

Members of the phyla *Verrucomicrobiota* (class *Verrucomicrobiae*) and *Bacteroidota* (class *Bacteroidia*) phyla are present in various environments, including soil and aquatic systems, such as the Baltic Sea [28,29]. They play an essential role in the degradation of complex organic matter, facilitating nutrient cycling and energy flow in ecosystems. Bacteria of the phylum *Verrucomicrobiota* were more abundant in water samples from the Kintai site, where they may regulate remineralization during the phytoplankton blooms [30]. Peaks of *Bacteroidota* in sediments, particularly in Melnragė in 2021, suggest localized enzymatic degradation activities [31].

*Firmicutes* (class *Bacilli*), although less abundant in water, showed a significant presence in sediments, particularly at the Šventoji site in 2021, which may indicate their role in sediment-specific processes such as the fermentation of organic matter [32].

The phylum *Campilobacterota* plays a role in the sulfur and nitrogen cycle [33]. It also includes the genus *Campylobacter*, which is often associated with pathogenic bacteria that can cause disease in humans and animals [34] and was detected in algal wrack accumulations along the Lithuanian coast [35]. In this study, *Campilobacterota* was the most abundant in both water (in 2017 and 2021) and sediment (in 2021 and 2023) samples at the Šventoji bathing site, where a high relative abundance of the same phylum was found in algal wrack accumulations affected by riverine outflow by another study [36].

The phylum *Desulfobacterota*, which was completely absent from Kintai and the Port water samples, was particularly abundant in sediments, highlighting its role in the anaerobic degradation of organic pollutants and the cycling of sulfur compounds in anoxic environments [37,38]. Similarly, the higher relative abundance of *Chloroflexi* in sediments compared to water samples suggests their involvement in sedimentary biogeochemical cycles, particularly in organic matter degradation and nutrient cycling [39].

*Planctomycetota* (class *Planctomycetes*) are commonly found in a variety of environments, including freshwater, marine, and terrestrial ecosystems, as well as in hotspots for the spread of AMR genes, such as wastewater treatment plants and clinical settings, as their presence correlates with ARGs [40].

### 3.2. Antibiotic Resistance Genes

A quantitative PCR approach utilizing a set of 384 primers was employed to investigate the environmental antibiotic resistomes along the Baltic Sea coast of Lithuania and the Curonian Lagoon. The total number of detected genes across different sampling sites ranged from 284 to 301, encompassing major classes of antibiotics, including aminoglycosides, beta-lactams, macrolide–lincosamide–streptogramin B (MLSB), phenicol, quinolones, sulfonamides, tetracyclines, trimethoprim, vancomycin, MDR, and other resistance genes, such as those associated with disinfectants and heavy metals. The findings of our study demonstrate a notable prevalence of ARG within the sampled coastal and lagoon sites. Of the identified ARGs, 50% were classified into three primary antibiotic groups: aminoglycosides, beta-lactams, MLSB, as well as MDR.

The aminoglycoside resistance genes *aadA7*, *aac(3)-iid_iia*, *aph4-ib*, *aac3-Iva*, *spcN*, and *aph3-ib* encode enzymes that modify aminoglycoside antibiotics, rendering them ineffective. These were among the most abundant ARGs detected in all sites of this study. The latter are classified as “current threat I” meaning that they have already been identified in pathogens [41]. Of particular concern among these aminoglycoside resistance genes is *aadA7*, which includes resistance to commonly used antibiotics such as streptomycin and spectinomycin [42]. It was the most abundant gene across the study sites. The remaining aminoglycoside ARGs encode resistance against other important antibiotics, including gentamicin (*aac(3)-iid_iia*), kanamycin and amikacin (*aph4-ib*), and tobramycin (*aac3-Iva*), and are frequently found on mobile genetic elements (*aph3-ib*) [43].

The genes *ermX_2*, *mphA*, *ereA*, *pncA*, and *ermE* encode resistance to macrolides, lincosamides, and streptogramin B antibiotics. The most commonly used MLSB antibiotics for treating respiratory and sexually transmitted infections are azithromycin and clarithromycin. Resistance to these antibiotics is encoded by the *mphA* gene (rank I) [44]. The *ereA* gene encodes an enzyme that hydrolyzes the lactone ring of erythromycin, thereby inactivating the antibiotic [45]. The present study revealed the presence of both the *mphA* and *ereA* genes in water and sediment samples collected from all study sites in 2017, 2018, 2021, and 2023. Furthermore, azithromycin, clarithromycin, and erythromycin residues were identified in the summer of 2017 in four Lithuanian coastal municipalities. The concentration of azithromycin, i.e., in the channel of Klaipėda Port, was found to be 37 ng/L, while clarithromycin and erythromycin were present at concentrations of 126.50 ng/L and 95.5 ng/L, respectively [5].

The four beta-lactam *blaOXY*, *blaOXY1*, *blaGOB*, *blaSFO*, and *penA* (rank II) genes encode beta-lactamases. These enzymes give bacteria resistance to beta-lactam antibiotics by hydrolyzing the antibiotic’s beta-lactam ring. Their presence in bacterial populations poses significant public health challenges [46]. It is of particular concern in healthcare settings that the *blaOXY* and *blaOXY1* genes are present in *Klebsiella* species, which are capable of causing severe infections. The relative abundance of *blaOXY* and *blaSFO* gene copies in proportion to the 16S rRNA gene in the water samples of this study appears to be higher (10^−2^) than in the urban aquatic recipients in Sweden (10^−4^) [12].

The multidrug resistance genes *mdtA* (classified as “future threat”—rank II—not yet found in pathogens), *oprD*, and *pbrT* are involved in the regulation of efflux systems, porin channel modifications, and resistance to heavy metals [47]. The *pbrT* gene, which encodes a protein involved in lead import, was detected in water samples from all study sites, whereas it was almost absent in sediment samples. Lead import helps bacteria to manage or detoxify heavy metals and survive in environments contaminated with lead. A previous study indicated elevated concentrations of metals (Pb, Cr, Cd, As) in the fine-grained organic-rich sediments of the studied area [48]. 

Notably, the *mcr1* gene, which encodes resistance to colistin, also known as the “last chance” antibiotic, a critical antibiotic for treating pneumonia, was detected in all study sites. The *mcr1* gene, initially identified as a plasmid carried by *Escherichia coli* isolated in China in 2011, has since been detected in seven pathogenic species across 31 countries [41,49]. In Lithuania, the presence of *mcr1* has been detected in migratory birds [50]. The S1 and 3 integron class genes and transposons of mobile genetic elements, which were identified at all study sites, may facilitate the spread of ARGs through horizontal gene transfer systems [15].

As the sites investigated in this work (excluding the Port area) are used for recreational activities (water sports, swimming, and fishing), the detection of specific genes considered to be related to anthropogenic pollution (e.g., *intI*, *sul1*, and *blaOXA*), as well as genes of clinical and veterinary relevance (beta-lactams, quinolones, and vancomycin) [12], and their relative abundance raise concerns about water quality and human health risks from exposure to microorganisms harboring these resistance genes. Furthermore, the results of this study indicate that the cumulative relative abundance of ARGs is higher in sediment samples than in water samples. It is known that the abundance of ARGs significantly correlates with the salinity properties of the ocean and river beach soil [51]. Furthermore, salinity can enrich the genotypes in sediments resistant to salt and antibiotics [52]. However, in this study, the sediment samples obtained from the lagoon sites, including the Port area and Kintai, which are freshwater sites, exhibited a distinctive antimicrobial profile that was not observed in brackish water locations. This suggests that sediments may serve as an accumulation zone for ARGs, as they do for other emerging pollutants [53] and especially in the lagoons [19,20].

## 4. Materials and Methods

### 4.1. Study Area and Sample Collection

**Figure 7:**
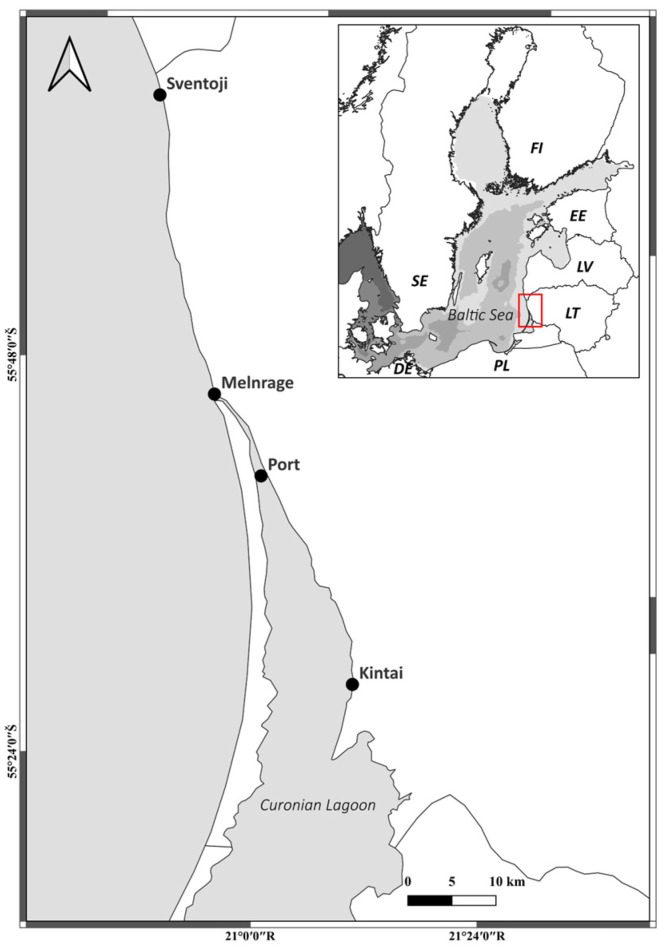
Map depicting study sites (black dots) along the Lithuanian coast of the Baltic Sea and the Curonian Lagoon (red box).

The geographical area under consideration in this study is situated on the southeastern coast of the Baltic Sea. From sixteen designated coastal bathing sites, two sites on the Baltic Sea coast (Šventoji and Melnragė), one Curonian Lagoon bathing site (Kintai), and a Port area (further as Port) (as the water exchange area between the lagoon and the Baltic Sea) were sampled (Figure 7). The Curonian Lagoon is Europe’s largest lagoon situated around the Baltic Sea, and it is primarily influenced by wind and the discharge of the Nemunas river. The freshwater outflow from the lagoon can be observed on beaches located to the north of the sole channel connecting the lagoon with the Baltic Sea, namely, the Klaipeda Strait, where the Port is allocated [25]. The salinity ranges from two to nearly seven practical salinity units (PSUs).

The surface water samples (50–60 cm) were collected monthly from June to September in 2017, 2018, 2021, and 2023. The samples were collected in sterile plastic bottles. Sediment samples were collected in 2021 (Šventoji and Melnragė) and 2023 (Šventoji, Melnragė, the Port area, and Kintai) using sediment core and placed in sterile plastic bags. All samples were transported in refrigerated containers at 4–8 °C and processed within four hours of collection.

Water temperature and salinity were measured in situ for all water samples each year using a handheld multiparameter probe (YSI Professional Series, YSI Incorporated, Yellow Springs, OH, USA). Total chlorophyll a (chl a) in the water samples was analyzed with FluoroProbe II, according to the accessory pigments.

All water samples (500–1000 mL) were filtered through 0.22 μm pore size mixed cellulose ester filters (MontaMil^®^ Membrane Filters, Frisenette ApS, Knebel, Denmark). Filters were stored at −80 °C until DNA extraction.

### 4.2. DNA Extraction and Preparation

Environmental DNA was extracted from water samples using PowerWater^®^ DNA Isolation Kit (MO BIO Laboratories, Inc., Carlsbad, CA, USA) while from sediment samples using PowerSoil^®^ DNA Isolation Kit (MO BIO Laboratories, Inc., Carlsbad, CA, USA), according to the manufacturer’s instructions. The concentration of extracted environmental DNA was measured using Qubit™ 3 Fluorometer (Life Technologies, Carlsbad, CA, USA). Aliquots were stored at −80 °C.

In this study, DNA aliquots were pooled for microbiome and resistome analysis using different strategies, assuming (i) cost-effectiveness compared to analysis of separate replicates and (ii) no loss of data quality [54]. The first DNA pooling strategy was to combine all DNA aliquots extracted from sediment and water samples collected at each sampling site throughout the study period. DNA samples pooled according to the first pooling strategy were used to screen the entire ARG profile in one of the sampling sites (more in Section 4.4). The second pooling strategy was to pool data per sampling site per year separately for sediment and water. It was used to analyze the entire microbiome (Section 4.3) and to screen 80 primers of ARG of veterinary and clinical importance (Section 4.4). Further details of the pooling scheme are provided in the Supplementary Figure S2.

### 4.3. Microbiome Analysis

16S rRNA data analysis. The composition of the bacterial community was determined by Full-Length Amplicon Metagenomics Sequencing (PacBio HiFI) by scanning the amplicons of the bacterial 16S rRNA gene. The PacBio was performed by Novogene Bioinformatics Technology Co., Ltd. (Cambridge, UK). Data were generated on the PacBio Sequel II system. The sequence data have been uploaded to the NCBI BioProject: PRJNA1162738.

The resulting demultiplexed PacBio fastq sequence files were processed using QIIME2 (2023.7 version) and FastQC to inspect the quality of the sequences based on the read quality scores [55,56]. To remove the PCR primers and possible remaining sequencing adapters, the Cutadapt plugin of qiime2 was used [57]. The denoising was performed using the dada2 plugin, which allows for constructing Amplicon Sequence Variants (ASVs) by noise reduction and deduplication at 100% similarity [58]. The ASVs were compared with the Silva 138 database at a 99% confidence level [59], using a Naive Bayes pre-trained classifier and the sklearn machine learning algorithm [60,61,62]. Relative abundance tables were generated at various taxonomic levels (kingdom to species). The ASV representative sequence tables obtained at the dada2 denoising step were conditionally filtered to retain the ASVs with a relative abundance of ≥1% in at least one of the samples. The Origin 2024b (10.1.5.132) software was used to generate the corresponding bar plots. The relative abundance data and the taxonomic assignations were used to perform statistical analyses, including alpha (Chao1 and Shannon index) and beta diversity metrics (PCoA based on Weighted UniFrac distance), to assess the community composition and relationship between samples, using a combination of R (version 4.3.2) and qiime2 diversity plugins.

### 4.4. High-Throughput Quantitative PCR Analysis

First, a total of 384 primer sets was used to screen the extended ARG profile in four pooled environmental DNA samples (sample pooling strategy #1) collected in the summer period from each sampling site (Šventoji, Melnragė, the Port, and Kintai) from different years (2017, 2018, 2021, and 2023) (Supplementary Figure S2). Selected primer sets target ARGs conferring resistance to nine major classes of antibiotics, including aminoglycosides (n = 60), beta-lactams (n = 54), macrolide–lincosamide–streptogramin B (MLSB) (n = 47), phenicol (n = 22), quinolone (n = 11), sulfonamide (n = 60), tetracycline (n = 26), trimethoprim (n = 17), vancomycin (n = 24), covering main antibiotic resistance mechanisms such as efflux pump, deactivation and cellular protection. These four pooled samples were also screened for multidrug resistance (MDR) (n = 39), integrons, mobile genetic elements (n = 52), and other genes (n = 17) encoding resistance to mercury, lead, bacitracin, colistin, antiseptics, etc. More details on target genes are provided in the Supplementary Table S2.

Later, 80 primer sets of veterinary and clinical importance [41] were selected and screened in the remaining samples (sample pooling strategy #2) for better temporal resolution: 13 seawater DNA samples from each sampling site at different sampling periods and six sediment DNA samples from each sampling site.

ARG detection and quantification were performed using the SmartChip^TM^ Real-Time PCR system (TakaraBio, Mountain View, CA, USA) by Resistomap Oy (Helsinki, Finland) as previously described [17].

### 4.5. Statistical Analysis

Descriptive and statistical analyses were performed using the XLSTAT (2023.3.1) and Origin 2024b (10.1.5.132) software. Before the analysis, the normality of variables was tested using the Kolmogorov–Smirnov test. When data deviated from the normal probability distribution, the non-parametric Kruskal–Wallis test was applied to compare spatial and temporal differences of the studied microorganisms. In contrast, the Mann–Whitney test for pair-wise comparison of two samples was applied to compare microorganism parameters between coastal and lagoon sites. Spearman correlation (rs) was used to define a statistically significant relationship between the ten most abundant bacterial classes, temperature, salinity, and chl a. Relationships between variables were visualized using Principal Component Analysis (PCA). Results were considered significant at a *p*-value less than 0.05.

## 5. Conclusions

This study represents the first quantitative assessment of antimicrobial profiles and bacterial community composition in the water and sediments of the Baltic Sea and the Curonian Lagoon. The observed differences in bacterial community composition between the Baltic Sea coast and the Curonian Lagoon demonstrate the influence of salinity and chl a concentration. The high prevalence of ARGs, particularly those linked to clinically important antibiotic classes, in both water and sediments emphasizes the need for monitoring of these environments. Furthermore, the distinct AMR profiles observed in the sediments of the Curonian Lagoon sites underscore the pivotal role of aquatic ecosystems, mainly sediments, in accumulating and disseminating antibiotic resistance genes. These findings reinforces the necessity to consider sedimentary environments in the broader context of mitigating the spread of antibiotic resistance in aquatic environments.

## Data Availability

The original contributions presented in the study are included in the article/Supplementary Material; further inquiries can be directed to the corresponding author/s.

## Supplementary Materials

The following supporting information can be downloaded at https://www.mdpi.com/article/10.3390/antibiotics13111013/s1. Table S1. Summary of alpha-diversity indices expressed in Chao1 and Shannon; Table S2. Summary of the targeted 384 genes examined in four sampling sites (pooled samples according to the pooling strategy #1), their relative abundance per 16S rRNA gene, and 80 genes’ primer set list (+) of veterinary and medical importance examined in samples from four study sites (Šventoji, Melnragė, Port, and Kintai) in different sample types (sediment and water) in different years (2017, 2018, 2021, and 2023) according to pooling strategy #2; Figure S1. Microbial community similarity (beta diversity) of samples expressed as PCoA of weighted UniFrac distances. The first two principal coordinates (PCs) explain 30.32% and 19.94% of the variance, respectively. Label deconstruction (W—water and S—sediment; Sv—Šventoji, M—Melnragė, P—Port, and Ki—Kintai; and the years 2017, 2018, 2021, and 2023); Figure S2. Sample pooling strategies used for microbiome and two different resolutions of resistome analysis. Pooling strategy #1 resulted in 4 environmental DNA samples; pooling strategy #2 resulted in 19 environmental DNA samples; Table S3. Descriptive statistics of the environmental parameters: salinity, temperature, and chlorophyll a (average ± standard deviation (SD)) at the investigated study sites.
