# Supplementary material for: Microbiome and Resistome Studies of the Lithuanian Baltic Sea Coast and the Curonian Lagoon Waters and Sediments
Source: Antibiotics (Basel). 2024 Oct 28;13(11):1013. doi: 10.3390/antibiotics13111013 (PMC11591088; doi:10.3390/antibiotics13111013)
Supplement: Supplementary file 1 [file antibiotics-13-01013-s001.zip › antibiotics-3245188-supplementary.pdf]

antibiotics

MDPI

Article

MICROBIOME AND RESISTOME STUDIES OF THE LITHUANIAN BALTIC SEA COASTAL AND THE CURONIAN LAGOON WATERS AND SEDIMENTS

Greta Gyraitė[1], Marija Kataržytė[2], Rafael Picazo Espinosa[2], Greta Kalvaitienė[2], and Eglė Lastauskienė [1]

1 Bioscience institute, Life Science Center, Vilnius University, Vilnius
2 Marine Research Institute, Klaipeda University, Klaipėda
* Correspondence: greta.gyraite@gmc.vu.lt

Supplementary material

Table S1. Summary of alpha-diversity indexes expressed in Chao1 and Shannon.

Sample type	Sample_Name	Year	Chao1	Shannon
Water samples	Šventoji	2017	286	7.24
		2021	385	7.5
		2023	281	6.56
	Melnragė	2017	346	6.88
		2018	361	7.29
		2021	303	7.05
		2023	369	7.77
	Port	2017	312	6.53
		2018	374	7.77
		2023	272	7.39
	Kintai	2017	288	7.27
		2018	251	6.88
		2023	310	7.46
Sediment samples	Šventoji	2017	262	6.78
		2023	432	8.12
	Melnragė	2021	367	7.94
		2023	451	8.48
	Port	2023	281	7.66
	Kintai	2023	230	7.21

Table S2. Summary of the targeted 384 genes examined in four sampling sites (pooled samples according to the pooling strategy #1), their relative abundance per 16S rRNA gene, and 80 genes primer set list (+) of veterinary and medical importance examined in samples from four study sites (Šventoji, Melnragė, Port, and Kintai) in different sample types (sediment and water) in different years (2017, 2018, 2021 and 2023) according to pooling strategy #2.

Gene group	Target genes tested in 384 primer set	Target genes tested in 80 primer set	Šventoji	Melnragė	Port	Kintai
**Aminoglycoside resistance**	*aadA7*	+	0.43427	0.58913	0.52365	0.56383
	aac(3)-iid_iia	+	0.09717	0.13397	0.10932	0.10319
	aph4-ib		0.06293	0.10271	0.08362	0.08657
	spcN		0.06352	0.10130	0.08657	0.07484
	aac3-IVa		0.05954	0.09321	0.06714	0.08171
	aph3-ib	+	0.05354	0.08921	0.07229	0.05725
	aph6-ia	+	0.06441	0.09440	0.04430	0.03640
	aac(6)-ir	+	0.03326	0.05007	0.03777	0.03751
	aadB	+	0.01946	0.02882	0.02882	0.02765
	aadA16		0.01924	0.02658	0.02325	0.02527
	aac(3)-xa_1		0.01933	0.02856	0.01577	0.01246
	aadA1_2	+	0.01381	0.02628	0.01475	0.01118
	aac(3)-ib		0.01118	0.02574	0.01541	0.01082
	aac(6′)-Ib_1	+	0.01287	0.01957	0.00872	0.00976
	ant6-ia	+	0.01210	0.01618	0.00971	0.00917
	aacC4		0.00583	0.00734	0.00978	0.01128
	aac(6)-iic		0.00622	0.00855	0.00995	0.00830
	aac(6)-iv_ih		0.00707	0.01003	0.00467	0.00405
	aac(6)-iz		0.00481	0.00829	0.00513	0.00321
	rmtB		0.00467	0.00678	0.00456	0.00397
	ant4-ib		0.00407	0.00602	0.00443	0.00444
	aadA10		0.00256	0.00422	0.00278	0.00242
	aac(3)-id_ie		0.00172	0.00402	0.00276	0.00266
	ant6-ib		0.00170	0.00305	0.00315	0.00225
	aph3-iii	+	0.00136	0.00342	0.00189	0.00194
	aadA5_2		0.00167	0.00304	0.00190	0.00152
	aadA9_1		0.00209	0.00250	0.00209	0.00145
	aadA2_1		0.00112	0.00202	0.00165	0.00207
	aac(6)-ig		0.00131	0.00235	0.00174	0.00118
	aadA6		0.00073	0.00099	0.00122	0.00104
	aac(6′)-Iy		0.00053	0.00100	0.00077	0.00062
	aadA2_3		0.00044	0.00073	0.00065	0.00080
	aadA_1		0.00045	0.00069	0.00096	0.00048

	aac(6)-is_iu_ix		0.00041	0.00115	0.00051	0.00045
	aph_viii		0.00064	0.00085	0.00055	0.00040
	armA_1		0.00040	0.00069	0.00032	0.00042
	aac6-aph2		0.00015	0.00081	0.00053	0.00032
	aph9-ia		0.00053	0.00088	0.00014	0.00019
	strB	+	0.00059	0.00049	0.00023	0.00024
	aac(6′)-II		0.00034	0.00034	0.00032	0.00033
	aph4-ia		0.00011	0.00045	0.00014	0.00006
	aadD		0.00022	0.00013	0.00012	0.00014
	aac(6)-im		0.00007	0.00023	0.00011	0.00010
	strA		0.00011	0.00019	0.00013	0.00005
	aphA3_1		0.00009	0.00014	0.00009	0.00008
	aph3-viia	+		0.00007	0.00014	0.00014
	aph3-via			0.00030		
	apmA			0.00015	0.00007	0.00007
	aph(3′′)-ia	+	0.00007	0.00007	0.00005	0.00006
	aacA43		0.00011		0.00011	
	aac(6′)I1			0.00009	0.00006	0.00004
	aacC2					
	aac(6)-iw					
	str					
	aac(6)-ij					
	armA_2					
	aadE					
	aphA1/7					
	aph(2′)-Ib					
	aacA/aphD					
**Beta-lactam resistance**	*blaOXY*		0.06515	0.09151	0.05968	0.06193
	blaGOB		0.03974	0.09672	0.05177	0.04289
	penA	+	0.03581	0.04565	0.04359	0.03865
	blaSFO		0.03716	0.03838	0.04318	0.04230
	blaOXY1		0.02739	0.03615	0.03483	0.04825
	blaACT		0.01415	0.01964	0.02015	0.02005
	blaMIR		0.01418	0.01454	0.01679	0.01655
	blaCTX-M	+	0.00960	0.01293	0.00819	0.00964
	blaOXA48	+	0.00498	0.01105	0.00598	0.00640
	blaOCH		0.00520	0.00803	0.00645	0.00631
	blaFOX		0.00403	0.00619	0.00548	0.00593
	cphA_1		0.00530	0.00798	0.00453	0.00382
	blaNDM	+	0.00149	0.00400	0.00577	0.00748
	blaCMY_2	+	0.00163	0.00531	0.00424	0.00493

	blaSHV11	+	0.00306	0.00388	0.00523	0.00380
	blaCARB		0.00224	0.00511	0.00382	0.00313
	blaROB	+	0.00391	0.00473	0.00302	0.00254
	beta_ccra		0.00198	0.00220	0.00269	0.00269
	blaACC		0.00166	0.00290	0.00244	0.00197
	blaMOX/blaCMY		0.00127	0.00294	0.00226	0.00159
	ampC/blaDHA		0.00115	0.00210	0.00177	0.00139
	cfiA		0.00097	0.00172	0.00143	0.00095
	blaPAO		0.00099	0.00104	0.00089	0.00062
	blaIMI		0.00076	0.00116	0.00078	0.00072
	imiR_2		0.00069	0.00106	0.00090	0.00066
	blaVIM	+	0.00076	0.00108	0.00076	0.00047
	blaPER		0.00087	0.00138		0.00073
	blaOXA51		0.00053	0.00079	0.00080	0.00076
	bla-L1		0.00033	0.00082	0.00074	0.00093
	blaCTX-M_5		0.00040	0.00091	0.00052	0.00043
	blaKPC	+	0.00026	0.00053	0.00042	0.00054
	ampC_cefa		0.00031	0.00043	0.00024	0.00035
	beta_B2		0.00017	0.00054	0.00032	0.00016
	pbp		0.00011	0.00042	0.00037	0.00019
	blaLEN		0.00022	0.00041	0.00022	0.00024
	blaTEM	+	0.00034	0.00021	0.00011	0.00023
	blaSME		0.00017	0.00026	0.00018	0.00020
	blaPSE		0.00010	0.00018	0.00030	0.00023
	blaBEL-nonmobile		0.00013	0.00028	0.00015	0.00014
	blaHERA		0.00012	0.00020	0.00010	0.00017
	blaB			0.00006	0.00011	0.00017
	cfxA		0.00007	0.00007		0.00006
	cepA		0.00005		0.00005	0.00006
	blaIND				0.00004	0.00004
	blaVEB					0.00003
	bl1acc					
	blaGES					
	blaCTX-M_8					
	blaTLA					
	blaZ					
	pbp5					
	mecA					
	bla1					
	blaADC-nonmobile					
**Integrons**	*intI1_1*	+	3.94949	4.14106	2.92817	2.36745

	intI3	+	0.50464	0.69737	0.62561	0.70385
	intI1_2		0.00024	0.00023	0.00038	0.00025
	intI2_2					
**Multidrug resistances (MDR)**	*oprD*		0.10584	0.12102	0.09583	0.09628
	mdtA	+	0.06919	0.09214	0.09921	0.11240
	pbrT	+	0.02970	0.04389	0.04239	0.04491
	mdtH	+	0.02843	0.04737	0.02889	0.02762
	emrD_1	+	0.01758	0.02708	0.02849	0.02592
	czcA	+	0.02475	0.03483	0.01791	0.01665
	sugE	+	0.02001	0.03040	0.02015	0.01750
	arsA	+	0.01421	0.02169	0.01402	0.01573
	acrR_1		0.01441	0.01854	0.01055	0.01019
	mdtE	+	0.00591	0.01010	0.01650	0.02052
	tolC_2	+	0.01176	0.01893	0.01070	0.00996
	mepA	+	0.01297	0.01770	0.01008	0.00821
	mexB	+	0.00960	0.01031	0.01096	0.00868
	ttgA		0.00771	0.01239	0.00951	0.00766
	acrF		0.00401	0.00682	0.00912	0.01252
	qacF/H	+	0.00769	0.00626	0.00227	0.00204
	cadC		0.00303	0.00503	0.00237	0.00297
	adeA		0.00161	0.00314	0.00330	0.00352
	qacA/B	+	0.00193	0.00225	0.00229	0.00210
	pcoA		0.00143	0.00352	0.00107	0.00106
	copA	+	0.00093	0.00225	0.00170	0.00215
	acrA_1		0.00129	0.00180	0.00164	0.00128
	mexA		0.00051	0.00196	0.00103	0.00091
	acrB_1		0.00095	0.00138	0.00074	0.00060
	terW		0.00042	0.00067	0.00100	0.00089
	mexE		0.00031	0.00041	0.00052	0.00050
	marR_3		0.00014	0.00027	0.00051	0.00066
	bexA/norM				0.00078	0.00022
	mdsA		0.00024	0.00032	0.00018	0.00019
	cefa_qacelta		0.00018	0.00020	0.00019	0.00023
	oqxA		0.00023	0.00034	0.00012	0.00010
	tcrB		0.00009	0.00024	0.00021	0.00013
	adeI		0.00005	0.00016	0.00011	0.00009
	mdtG_1					
	cmr					
	mtrE					
	emrB/qacA_1					
	cfr					

	pmrA					
**Mobile genetic elements (MGE)**	*IS1111*		0.35684	0.46976	0.37458	0.34788
	orf37-IS26		0.23326	0.35849	0.16570	0.13934
	trbC		0.16743	0.23407	0.17657	0.14779
	Tn5403		0.13679	0.19981	0.15749	0.17678
	IS1247_1		0.11034	0.14293	0.11136	0.09583
	IS1133		0.07802	0.10732	0.09172	0.07748
	IS1247_2		0.13902	0.10319	0.03565	0.01229
	IS630		0.03404	0.04029	0.03467	0.03147
	ISCR1		0.03492	0.04523	0.02970	0.02998
	IS21-ISAs29		0.02246	0.03983	0.03691	0.03404
	tnpA_3		0.01619	0.02797	0.02010	0.01926
	EAE_05855		0.00567	0.02225	0.00458	0.00429
	IncI1_repI1		0.00532	0.00901	0.00866	0.00553
	IncP_oriT		0.00535	0.00721	0.00652	0.00605
	IncW_trwAB		0.00436	0.01120	0.00463	0.00340
	IncN_rep		0.00372	0.00690	0.00371	0.00261
	cro		0.00067	0.00105	0.00226	0.00735
	ISEcp1		0.00467	0.00321	0.00155	0.00186
	ISPps		0.00050	0.00140	0.00708	0.00211
	IS613		0.00195	0.00222	0.00358	0.00312
	IS3		0.00158	0.00258	0.00247	0.00188
	Tp614		0.00159	0.00224	0.00222	0.00127
	IncQ_oriT		0.00171	0.00257	0.00137	0.00146
	traN		0.00149	0.00085	0.00107	0.00142
	IS6100		0.00097	0.00131	0.00122	0.00128
	IS5/IS1182		0.00140		0.00122	0.00164
	tnpA_2		0.00035	0.00077	0.00097	0.00073
	IS200_2		0.00032	0.00039	0.00076	0.00041
	trfA		0.00010	0.00041	0.00038	0.00080
	tnpA_7		0.00026	0.00066	0.00040	0.00028
	lncF_FIC		0.00029	0.00046	0.00021	0.00012
	Tn3		0.00014	0.00029	0.00043	0.00017
	Tn5		0.00018	0.00051	0.00019	0.00009
	tnpA_4		0.00012	0.00012	0.00013	0.00040
	tnpA_1		0.00015	0.00025	0.00008	0.00014
	IS6/257		0.00009	0.00013	0.00021	0.00018
	IS200_1		0.00015	0.00011	0.00013	0.00021
	tnpA_5		0.00053			
	IS26_1		0.00006	0.00014	0.00012	0.00014
	IncHI2-smr0018		0.00013		0.00014	0.00009

	tnpA_6		0.00009		0.00008	0.00005
	ISAba3			0.00018		0.00005
	pAKD1					0.00005
	ISEfm1					
	IncN_oriT					
	IS256					
	pAMBL					
	IncN_korA					
**Macrolide-. lincosamide- and streptogramins B (MLSB) resistance**	*ermX_2*	+	0.11085	0.17194	0.17274	0.17076
	mphA	+	0.10857	0.19211	0.14593	0.13366
	ereA	+	0.07856	0.08778	0.09004	0.12616
	pncA		0.05280	0.07856	0.07097	0.06352
	ermE		0.04596	0.06576	0.05378	0.04949
	ermO		0.01338	0.01370	0.01908	0.01873
	erm35		0.00785	0.01240	0.00904	0.01187
	mefA	+	0.00774	0.00888	0.00805	0.00937
	erm42		0.00520	0.01160	0.00694	0.00827
	oleC		0.00653	0.00989	0.00527	0.00319
	erm36		0.00321	0.00734	0.00726	0.00642
	vat(B)		0.01237	0.00588	0.00265	0.00178
	carB		0.00256	0.00319	0.00344	0.00293
	vat(A)	+	0.00220	0.00280	0.00243	0.00215
	vgaB_1		0.00134	0.00291	0.00194	0.00171
	ermD/K		0.00070	0.00123	0.00156	0.00207
	mphB	+	0.00128	0.00158	0.00089	0.00129
	mefA_1		0.00102	0.00152	0.00083	0.00139
	vgaA_1		0.00099	0.00083	0.00133	0.00103
	ermB_3	+	0.00028	0.00043	0.00070	0.00073
	ermA		0.00038	0.00068	0.00054	0.00048
	ermD		0.00038	0.00067	0.00033	0.00034
	ermX_1		0.00043	0.00044	0.00046	0.00038
	lsaC		0.00038	0.00044	0.00048	0.00039
	lnuF		0.00016	0.00039	0.00025	0.00021
	ermB_2		0.00012	0.00055	0.00009	0.00023
	mefB		0.00009	0.00021	0.00035	0.00019
	ermD_1		0.00016	0.00033	0.00010	0.00014
	lmrA_1		0.00029	0.00017	0.00012	0.00007
	erm34		0.00016	0.00023	0.00009	0.00008
	vatE_2	+	0.00012	0.00010	0.00011	0.00010
	msrC_1		0.00015	0.00008	0.00005	0.00009
	vga(A)LC_1		0.00008	0.00011	0.00010	0.00007

	ermF		0.00010		0.00004	0.00005
	lnuC					0.00005
	msrD					
	ermA/ermTR					
	ermF_1					
	msrA_1					
	ermY					
	lnuB					
	ermC_2					
	msrE					
	ermB_1					
	ermT_1					
	lnuA_1					
	pikR2					
**Others**	*bacA*	+	0.04825	0.06207	0.04949	0.05447
	mcr1	+	0.01253	0.01176	0.00656	0.00462
	arr3		0.00176	0.00319	0.00208	0.00270
	ttgB		0.00086	0.00175	0.00244	0.00184
	merA		0.00099	0.00175	0.00156	0.00153
	nisB_1	+	0.00109	0.00176	0.00132	0.00140
	fabK	+	0.00091	0.00134	0.00127	0.00086
	arr2		0.00059	0.00151	0.00071	0.00071
	fosb		0.00036	0.00047	0.00051	0.00064
	nimE	+	0.00022	0.00042	0.00023	0.00025
	fosX		0.00018	0.00021	0.00019	0.00042
	qacEâˆ†1_3	+	0.00005	0.00012	0.00010	0.00009
	qacEâˆ†1_1					0.00003
	crAss56					
	crAss64					
	sat4					
	mcr2					
**Phenicol resistance**	*cmlV*		0.02314	0.03883	0.02856	0.02441
	floR_1	+	0.00856	0.01097	0.01294	0.01382
	cmxA		0.00493	0.00897	0.00785	0.00719
	catA3	+	0.00239	0.00426	0.00428	0.00379
	cmlA_2	+	0.00304	0.00381	0.00237	0.00304
	ceoA		0.00218	0.00418	0.00276	0.00215
	cat		0.00214	0.00344	0.00266	0.00242
	yidY/mdtL		0.00279	0.00282	0.00223	0.00273
	floR		0.00342	0.00207	0.00201	0.00213
	catP		0.00063	0.00131	0.00106	0.00143

	optrA		0.00108	0.00133	0.00093	0.00075
	catB9	+	0.00139	0.00103	0.00068	0.00066
	cat(pC221)		0.00029	0.00101	0.00035	0.00041
	catB8		0.00030	0.00036	0.00026	0.00032
	catB3		0.00027	0.00033	0.00029	0.00017
	mdtL	+	0.00018	0.00043	0.00029	0.00009
	cmlA_4		0.00011	0.00015	0.00010	0.00008
	catA2		0.00010	0.00007	0.00012	0.00011
	catA1					
	catB2					
	fexA					
	catQ					
**Quinolone resistance**	*qepA*	+	0.11798	0.17434	0.15496	0.15248
	qnrB4		0.05886	0.09151	0.04328	0.03557
	qnrB	+	0.00425	0.00407	0.00291	0.00199
	qnrS2		0.00248	0.00342	0.00285	0.00265
	qnrB_2		0.00201	0.00466	0.00214	0.00197
	qnrD		0.00110	0.00159	0.00106	0.00128
	qnrVC_2		0.00059	0.00070	0.00114	0.00115
	norA	+	0.00068	0.00124	0.00040	0.00043
	qnrVC1_VC3_VC6		0.00041	0.00077	0.00052	0.00049
	qnrA	+	0.00022	0.00021	0.00012	0.00012
	qnrS_1	+	0.00006	0.00007	0.00012	0.00013
**Sulfonamide resistance**	*sul3_1*		0.01057	0.02100	0.01373	0.01795
	sul2_2	+	0.01302	0.01516	0.00971	0.00609
	sul4		0.00174	0.00377	0.00268	0.00259
	sul1_2	+	0.00035	0.00078	0.00024	0.00034
	folP_2		0.00010	0.00033	0.00023	0.00018
	folA_1		0.00005	0.00009	0.00013	0.00015
**Tetracycline resistance**	*tetD*	+	0.08617	0.10463	0.07966	0.07856
	tetG		0.07313	0.07450	0.06714	0.06776
	tetL_2	+	0.03089	0.05581	0.03657	0.03919
	tetR		0.01083	0.01726	0.01592	0.01209
	tetA_2	+	0.00329	0.00463	0.00403	0.00318
	tetM	+	0.00104	0.00910	0.00181	0.00175
	tetJ		0.00124	0.00292	0.00219	0.00273
	tetA/B_1		0.00067	0.00166	0.00088	0.00138
	tetR_1		0.00026	0.00061	0.00090	0.00058
	tetE		0.00053	0.00073	0.00026	0.00029
	tet36_1		0.00009	0.00010	0.00023	0.00026
	tetC_2	+	0.00016	0.00012	0.00005	0.00012

	tetW	+		0.00013	0.00012	0.00016
	tet44		0.00008	0.00008	0.00011	0.00007
	tetS		0.00013	0.00018	0.00004	
	tet38		0.00009	0.00009	0.00007	0.00008
	tetPB_1					0.00004
	tetX					
	tet39					
	tetH					
	tetT					
	tet32					
	tetK					
	tetQ					
	tetO_2					
	tetPA					
**Trimethoprim resistance**	*dfrA7*		0.01614	0.02655	0.01673	0.01091
	dfrA27		0.00874	0.01415	0.00813	0.00607
	dfrA25	+	0.00396	0.00617	0.00604	0.00627
	dfrA22		0.00213	0.00399	0.00335	0.00374
	dfra21		0.00232	0.00420	0.00269	0.00269
	dfrB	+	0.00354	0.00346	0.00324	0.00126
	dfrA8		0.00057	0.00130	0.00103	0.00244
	dfrA15	+	0.00040	0.00071	0.00035	0.00034
	dfrA12	+	0.00032	0.00036	0.00037	0.00026
	dfrA10		0.00026	0.00036	0.00023	0.00024
	dfrA1_1	+		0.00022	0.00010	0.00023
	dfrK			0.00017		0.00004
	dfra17					0.00003
	dfrG					
	dfrA19					
	dfrAB4					
	dfrC					
**Vancomycin resistance**	*vanTC_2*		0.06983	0.09539	0.09300	0.09354
	vanA	+	0.02524	0.07948	0.05219	0.05479
	vanHB		0.01249	0.01820	0.01252	0.00991
	vanYD_1	+	0.00518	0.01140	0.00822	0.00793
	vanB_1	+	0.00252	0.00576	0.00505	0.00421
	vanRB		0.00151	0.00274	0.00175	0.00163
	vanTE		0.00089	0.00132	0.00188	0.00162
	vanC2		0.00092	0.00175	0.00089	0.00190
	vanYB	+	0.00081	0.00119	0.00097	0.00100
	vanSC_2		0.00092	0.00084	0.00042	0.00044

	vanC_2		0.00025	0.00038	0.00013	0.00067
	vanHD		0.00021	0.00051	0.00030	0.00034
	vanWB			0.00008	0.00007	0.00018
	vanTG		0.00006		0.00018	0.00006
	vanRC			0.00018		
	vanG			0.00007		0.00004
	vanXB					0.00005
	vanSB					0.00004
	vanD					
	vanRD					
	vanRC4					
	vanRA_1					
	vanSA					
	vanXA					

Figure S1. Microbial community similarity (beta diversity) of samples expressed as PCoA of weighted UniFrac distances. The first two principal coordinates (PC) explain 30.32% and 19.94% of the variance, respectively. Label deconstruction (W - water and S - sediment; Sv - Šventoji, M - Melnragė, P - Port, Ki - Kintai; and the years 2017, 2018, 2021 and 2023).

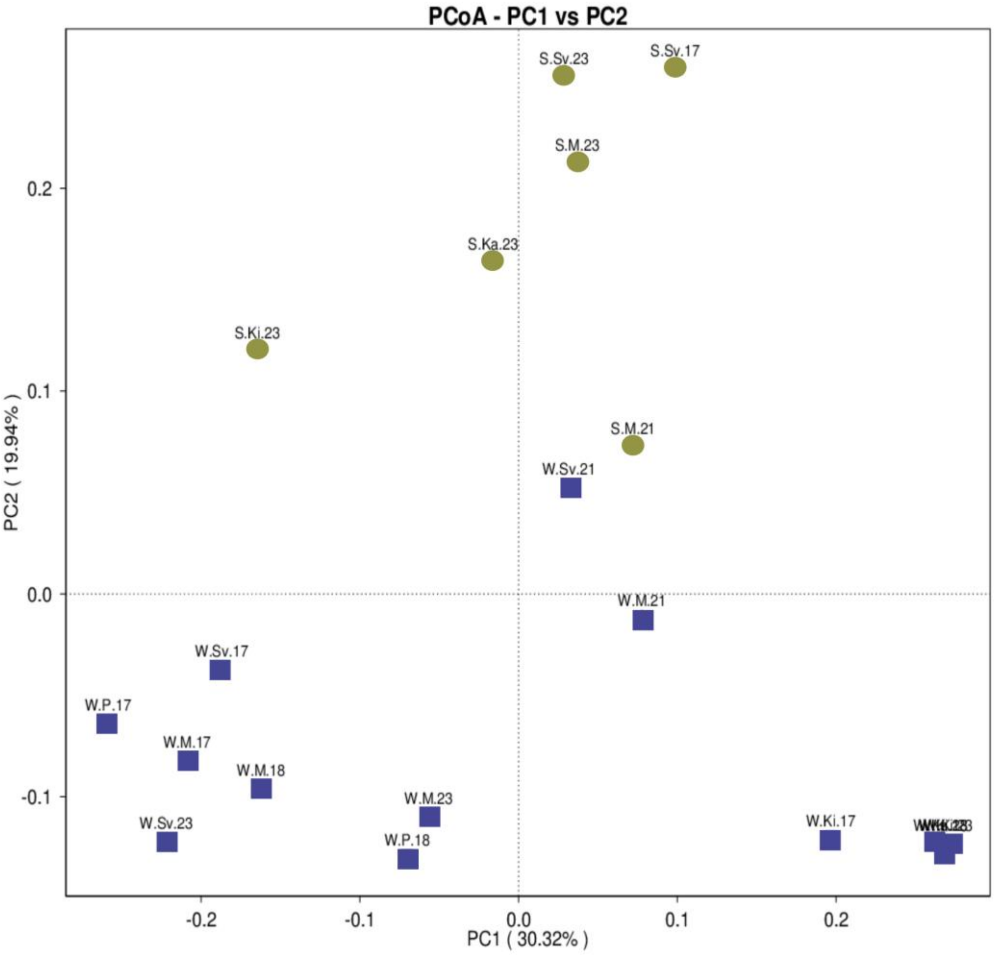

Figure S2. Sample pooling strategies used for microbiome and two different resolutions of resistome analysis. Pooling strategy #1 resulted in 4 environmental DNA samples; pooling strategy #2 resulted in 19 environmental DNA samples.

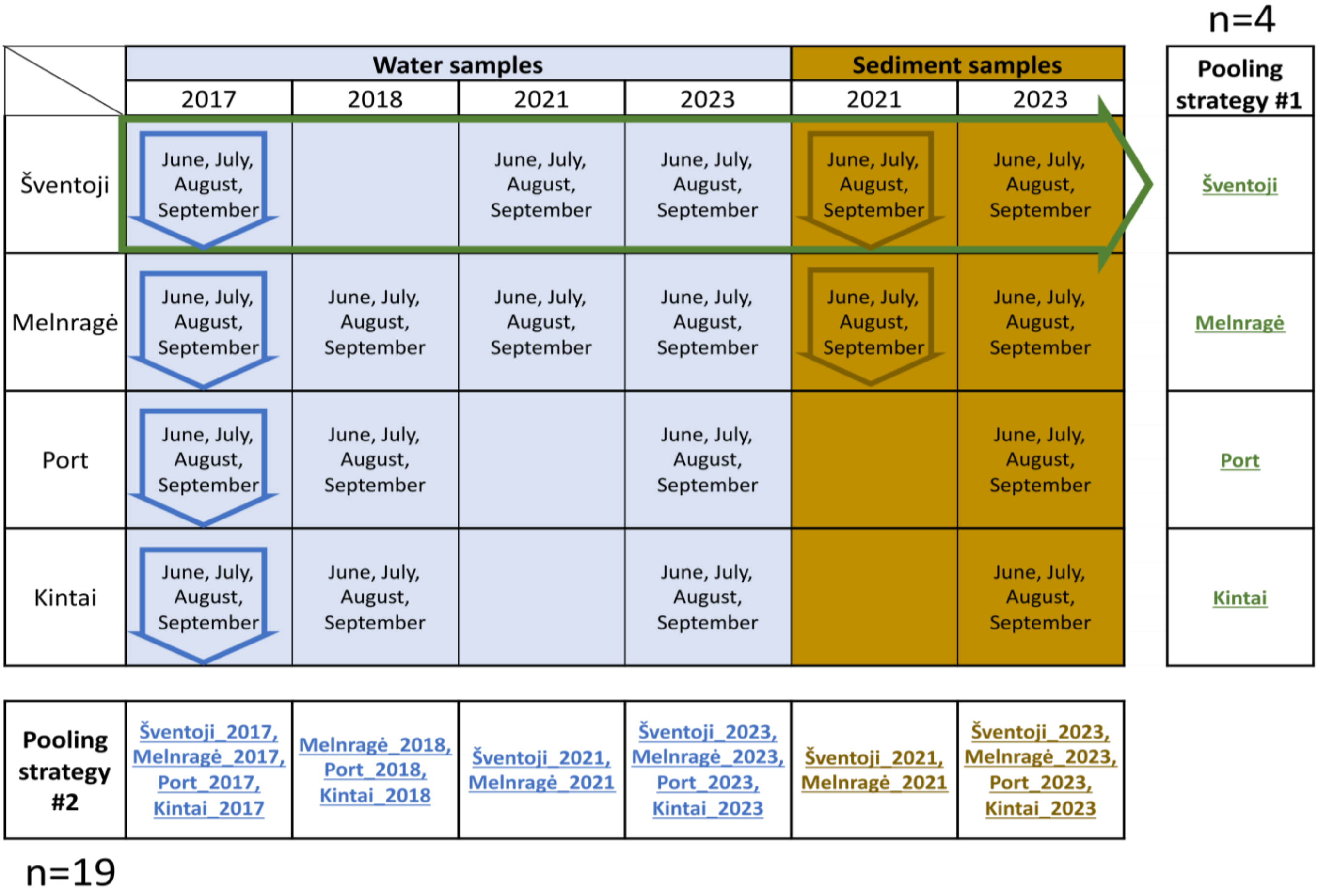

Table S3. Descriptive statistics of the environmental parameters: salinity, temperature and chlorophyll a (average ± standart deviation (SD)) at the investigated study sites.

Site	Year	Salinity. PSU (Average±SD)	Temperature. ºC (Average±SD)	Chlorophyll a. ug/L^{-1} (Average±SD)
Šventoji	**2017**	5.18±1.08	17.80±1.73	16.77±3.46
	**2021**	6.09±0.49	18.43±3.53	20.03±2.84
	**2023**	6.45±0.28	17.50±3.23	21.14±4.91
Melnragė	**2017**	5.14±0.99	18.10±1.60	12.89±2.73
	**2018**	5.72±1.72	18.97±2.95	32.22±25.48
	**2021**	5.72±0.98	16.87±2.81	19.83±5.08
	**2023**	5.05±0.93	18.18±1.91	13.99±5.13
Port	**2017**	3.66±2.07	18.73±1.40	24.55±6.26
	**2018**	4.27±2.49	20.31±3.91	21.59±8.64
	**2023**	1.23±0.98	20.35±1.82	9.72±1.83
Kintai	**2017**	0.27±0.06	17.25±0.82	12.70±7.19
	**2018**	0.79±0.73	20.53±3.42	5.62±9.46
	**2023**	0.49±0.48	20.08±2.21	11.06±11.06
